# Therapeutic potential of induced iron depletion using iron chelators in Covid-19

**DOI:** 10.1016/j.sjbs.2021.11.061

**Published:** 2021-12-13

**Authors:** Punnoth Poonkuzhi Naseef, Muhammed Elayadeth-Meethal, K.T. Mohammed Salim, A Anjana, C Muhas, K. Abdul Vajid, Mohamed Saheer Kuruniyan

**Affiliations:** a Department of Pharmaceutics, Moulana College of Pharmacy, Perinthalmanna 679321, Kerala, India; b Department of Animal Breeding and Genetics, College of Veterinary and Animal Sciences, Kerala Veterinary and Animal Sciences University, Pookode, Wayanad, 673576, Kerala, India; c Department of Pharmacy Practice, Al Shifa College of Pharmacy, Malappuram, 679325, Kerala, India; d Department of Pharmacy Practice, KTN College of Pharmacy, Chalavara, Palakkad, 679505, Kerala, India; e Department of Dental Technology, College of Applied Medical Sciences, King Khalid University, Abha 61421, Saudi Arabia

**Keywords:** COVID 19, Hyperferritinemia, Pathogenesis, Genomics, Proteomic interaction, Iron Chelator, Ferritin, Clinical use

## Abstract

Ferritin, which includes twenty-four light and heavy chains in varying proportions in different tissues, is primarily responsible for maintaining the body's iron metabolism. Its normal value is between 10 and 200 ngmL^−1^ in men and between 30 and 300 ngmL^−1^ in women. Iron is delivered to the tissue via them, and they act as immunomodulators, signaling molecules, and inflammatory markers. When ferritin level exceeds 1000 µgL^-1^, the patient is categorized as having hyperferritinemia. Iron chelators such as deferiprone, deferirox, and deferoxamine are currently FDA approved to treat iron overload. The inflammation cascade and poor prognosis of COVID-19 may be attributed to high ferritin levels. Critically ill patients can benefit from deferasirox, an iron chelator administered orally at 20–40 mgkg^−1^ once daily, as well as intravenous deferoxamine at 1000 mg initially followed by 500 mg every 4 to 12 h. It can be combined with monoclonal antibodies, antioxidants, corticosteroids, and lactoferrin to make iron chelation therapy effective for COVID-19 victims. In this article, we analyze the antiviral and antifibrotic activity of iron chelators, thereby promoting iron depletion therapy as a potentially innovative treatment strategy for COVID-19.

## 1 Introduction

Iron is one of the most important components of hemoproteins and is essential for oxygen transport in the electron transport chain and hepatic metabolism in cytochrome oxidase. A physiologic iron level within the normal range is crucial to prevent disruptions in oxygen transportation, oxidative phosphorylation, and subsequent metabolic impairments (Kernan and Carcillo, 2017). The amount of serum ferritin is an optimal diagnostic marker for iron overload or iron deficiency, as its proportion is modulated by the intracellular iron pool. Hyperferritinaemia-cataract syndrome often results from elevated serum ferritin levels without an erythrogenic response. The four main immune-directed diseases associated with high ferritin levels are macrophage activation syndrome (MAS), adult-onset Still's disease (AOSD), catastrophic antiphospholipid syndrome (CAPS) as well as septic shock. As these syndromes involve similar laboratory, clinical, and therapeutic manifestations, hyperferritinemia is likely a contributing factor to the development of these conditions (Colafrancesco et al., 2020).

Globally, COVID-19, which is caused by SARS-CoV-2, has posed a serious threat to humans. In COVID-19 patients, having an elevated serum ferritin concentration of more than 300 µgL^-1^ nine-fold increased the risk of death (Merad and Martin, 2020). These markers are used to identify the severity of the hyperferritinemia conditions and their relationship to COVID-19. Due to its typical immunomodulatory properties, this condition is associated with a variety of immune-mediated diseases (Para et al., 2021). The most commonly used iron-chelating agents in treating hyperferritinemia are deferiprone; deferoxamine, and deferasirox. Iron chelators have been shown to significantly enhance iron chelation and catabolize ferritin, whilst concurrently decreasing macrophage-derived cytotoxicity and supplementing antioxidant capacity, which could be beneficial in alleviating COVID-19 structured pathology (Swain and Romano, 2021).

In addition to understanding their importance in hyperferritinemia treatment, we aimed to determine how they might be used to decrease COVID 19 mortality. The purpose of our presentation was to encourage clinicians to consider iron chelation therapy as a strategy for managing COVID-19 and its secondary complications. An iron chelation treatment program aimed at improving the clinical outcomes in patients with COVID-19 can hasten advancements in the scientific field.

## 2 Basics of serum ferritin

The iron stored in serum ferritin is a biological form of iron that prevents the inherent toxicity of the iron metal from harming DNA, lipids, and proteins. Their role involves executing the functional lead in malignancy, inflammatory and cerebral disease (Saito, 2019). The shell is shaped like a sphere and contains twenty four subunits of light [L] and heavy [H] chains. Within its central cavity, 4,500 iron atoms are found in oxidized form. Ferroxidase activity is only found in the H subunit (Heggland et al., 2019). Many in vitro studies identify L-ferritin as a factor in iron incorporation, but recent research shows that L-ferritin has a stimulatory effect on cell proliferation, independent of iron availability (Ruscitti et al., 2020). The proportion of L or H subunits varies with the cell type and biological status, with a high concentration of H-subunits found in the heart and kidneys and a high concentration of L-subunits found in the liver and spleen (Kappert et al., 2020, Ding et al., 2017).

Ferritin synthesis is regulated by cytokines at all stages of gene expression, including cell division, expansion, inflammation, and integration (Cappanera et al., 2021). The residual tissue receptors are embodied to bind only the H-ferritin, unlike those expressed on hepatic cells which bind both L and H subunits (Bou-Abdallah et al., 2018). During laboratory animal experiments, it was determined that H-chain phagocytosis receptors were on mucin domain TIM-2 and T-cell immunoglobulins within kidney, liver, and B and T lymphocyte cells (Rietz et al., 2021). Recent studies have confirmed that Scara5 is a scavenger that can act on a variety of substrates, favorably L-ferritin, as opposed to TIM-2 (Yu et al., 2020).

### 2.1 Ferritin as a prognostic aid

Iron-overload conditions and conditions resulting from an iron-deficiency anemia are valuable indications of iron status, which can be evaluated with ferritin test. A particular example refers to hereditary hemochromatosis and transfusional overload. Blood routine tests are frequently recommended to confirm and treat those conditions, including serum value tests. As well as indicative of infrequent inflammatory disorders, elevated iron could also indicate diseases such as herediary hemophagocytic syndrome and Still's disease (Guo et al., 2017). For women, the normal range is 10–200 ngmL^−1^, while for men the normal range is 30–300 ngmL^−1^ (DePalma et al., 2021). When an iron level falls below 12 ngmL^−1^, the body has exhausted its iron reserves, and when it reaches 3000 ngmL^−1^ or greater, there has been liver injury in conjunction with iron overload.

## 3 Ferritin in physiological and pathological process

### 3.1 Ferritin as iron delivery system

It is important to note that the main site for iron storage in a living cell has a high capacity for holding iron in its ferric state (Fe^3+^). In its core, the molecule can contain about 4500 ferric atoms, and can therefore be used as a potentially effective iron delivery system (Conway and Henderson, 2019). Despite the fact that transferrin receptors are the primary process of iron uptake in erythroid cells, it was also demonstrated that macrophages can act as a reservoir of iron in hematopoietic prototype cells through ferritin release (Richard and Verdier, 2020). According to Sibille *et al.* (1998) there is the possibility of over 160,000 iron molecules being accumulated in one hepatocyte per minute (Sibille et al., 1988).

### 3.2 Ferritin as an immunomodulator

H-ferritin exerts immunomodulatory effects by blocking the generation of antibodies by B lymphocytes, preventing the delayed type of hypersensitivity, inhibiting granulocyte hyperendocytosis, and regulating granulomonocytopoiesis (Sottile et al., 2019). The cytokine IL-10 is produced by lymphocytes to suppress immune response (21). Based on available evidence, ferritin H-subunits function as critical components of receptor-mediated cell movement and signaling by chemokine receptors (Moreira et al., 2020).

### 3.3 Ferritin as signaling molecule

Shen et al. (Shen et al., 2021) suggested that exogenous ferritin plays a role in signaling in stellate hepatocytes. In contrast to its traditional role as a ferrous storage molecule, its ancillary role was entirely detached from its iron content in this study (Shen et al., 2021).

### 3.4 Ferritin as an inflammatory marker

Various conditions, including rheumatoid arthritis, chronic kidney disease, severe infection, and malignancy, would elevate the acute and chronic inflammatory indicator. A lack of iron in inflammatory conditions, conventionally identified as anemia of malignancies and inflammation, is a defense mechanism that prevents tumors and pathogens from utilizing serum iron.

## 4 Hyperferritinemia

Hyperferritinemia refers to an excess of ferritin in the body. There was a lack of consensus on how to interpret these results and the presence of more than 1000 µgL^-1^ was considered non-specific (Gómez-Pastora et al., 2020). A value exceeding 10,000 µgL^-1^ is considered extreme. The level of transferrin saturation, however, can serve as a valuable tool for identifying iron overload. Transferrin's iron-binding sites are estimated to be a proportion of its total number of iron-binding sites (Daude et al., 2020). Ferritin levels higher than 300 µgL^-1^ are present in about 20% of caucasian men irrespective of age. Age-wise changes in ferritin distribution are most noticeable during menstruation and pregnancy. A ferritin value over 200 µgL^-1^ is present in 3% of females aged 30 to 50 years, and the incidence increases with age (Senjo et al., 2018).

### 4.1 Underlying causes of hyperferritinemia

The diagnosis of hyperferritinemia in several inflammatory, infectious, and malignant conditions is essential to management, treatment, and prognosis. An abnormal ferritin level is present in the metabolic syndrome, obesity, insulin resistance or diabetes mellitus, excessive alcohol consumption and immune-mediated syndromes like hemophagocytic lymphohistiocytosis (HLH) and Still's disease. There are a few factors that contribute to hyperferritinemia caused by iron overload, such as hemochromatosis, dysmetabolic iron overload syndrome, and iron-loading anemias (Ruiz-Ordoñez et al., 2021, Cullis et al., 2018). Among the other causes of hyperferritinemia that lead to an iron overload or hyperferritinemia with iron overload are chronic liver diseases which include viral hepatitis, cirrhosis, alcoholic liver disease, and cutanetate dermatitis.

### 4.2 Ferritin in malignancy

The increase of ferritin in malignancy (300 to 1000 µgL^-1^) is associated with the displacement of ferritin constitution to further H-chain enriched strains (Cullis et al., 2018, Yamashita et al., 2017, Naymagon et al., 2020). The cytosol extract of benign breast carcinoma tissues demonstrated a 10-fold increase in the tissue ferritin content, which illustrated a substantial amount of ferritin in the non-benign epithelium, and sparse amounts in benign tissue.

### 4.3 Ferritin in chronic renal impairment

It was found that serum ferritin is an inferior bioavailability marker for iron in chronic renal impairment subjects (Ueda and Takasawa, 2018). The fact that about half of all hemodialysis patients have a value greater than 500 µgL^-1^ does not indicate a bioavailable iron for erythropoiesis, but inflammation may be the underlying factor (Balla et al., 2019). Moreover, serum ferritin levels over 800 g/L were associated with a higher malnutrition-inflammation score (Garg et al., 2018).

### 4.4 Ferritin in systemic inflammatory conditions

Study results showed that 6.7% of the study subjects had ferritin levels above 1000 µgL^-1^, which was associated with hepatic disease, sickle cell syndrome, HIV infection, kidney disease, and chronic transfusion. Patients on chronic transfusions and patients with sickle cell disease had the highest levels of iron. A hemophagocytic index of 45,000 µgL^-1^ suggests severe hemophagocytosis (Lee and Means, 1995). There was significant accuracy in predicting the extent of chronic disease among patients with Adult-Onset Still's Disease (AOSD) by using an extravagant response level of five times the average top level (Otrock et al., 2017).

### 4.5 Ferritin in hemophagocytic lymphohistiocytosis

A ferritin level above 500 µgL^-1^indicates hemophagocytic lymphohistiocytosis (HLH) or hemophagocytic syndrome (HPS). Basically, it consists of a wide range of inherited or acquired disorders characterized by hyperbolized and fatal inflammatory reactions (39). Hyperferritinemia is attributed to macrophage activation in acute macrophage activation syndrome (Henderson and Cron, 2020).

### 4.6 Ferritin in hemochromatotic hepatic impairment

Alcoholic patients with hepatic damage have higher levels of serum ferritin (Milman et al., 2019). An escalating iron level accompanies viral Hepatitis C infection. An iron deficiency has been established as a critical co-morbidity in viral hepatitis disease progression and advancement to liver failure (Czaja, 2019). The serum ferritin concentration in hepatitis C patients, especially those on hemodialysis, indicated severe hepatic impairment (Rujeerapaiboon et al., 2021). The ferritin range is being evaluated as part of sequential liver biopsy investigation and therapy (Buzzetti et al., 2019).

### 4.7 Ferritin in hereditary hyperferritinemia with congenital cataracts

Hereditary hyperferritinemia with congenital cataract is a rare autosomal dominant genetic disease characterized by the presence of pre-existing cataract and persistently elevated ferritin levels in blood plasma (1000 to 5000 gL^-1^). In this genetic disorder, the L-ferritin gene coding for a protein in the lens membrane is mutated.

## 5 Iron chelation therapy

Iron chelation therapy reduces iron overload due to its disposition in different organs such as the heart and liver after chronic transfusion and is recommended with ferritin level more than 1000 µgL^-1^. Deferasirox, deferiprone and deferoxamine are the most distinct US FDA authorized agents. Each of them has its benefits and drawbacks, various disease targets and amount of iron deposition which may alter their choice. Effective chelation confined with labile plasma iron and non-transferrin bound iron aid to avert adverse complications of iron overload and eliminate them through feces and urine (Aydinok, 2018, Nuñez and Chana-Cuevas, 2018).

### 5.1 Ideal characteristics

Compared to ferritin or transferrin, Chaberek and Martell state that ligands must have a higher affinity for iron element, and should have a lower affinity for other physiologically important cations than Fe (III) or Fe (II) (Barnard., xxxx). Secondly, the compound must be highly bioavailable. Third, the agent should be able to withstand enzymatic and hydrolytic degradation before and after absorption. It is also important for the chelator to be biocompatible without causing side effects (Barnard., xxxx).

### 5.2 Mechanism of action

Removal of cell iron depends on membrane permeability and the ability to compete with constituents of cell iron (Ghosh and Ghosh, 2018). Clinically, most chelators are ineffective in removing proteins that contain [Fe-S] clusters or heme (Sheikh et al., 2021). However, some chelators can offer significant inhibition to enzymes that require a constant supply of Fe-like ribonucleotide reductase (Amano et al., 2020), or enzymes containing mono- or di-Fe centers coordinated to oxygen ligands (Sheikh et al., 2021).

### 5.3 Deferasirox

Iron chelator that has a notable affinity for iron and a lesser affinity for calcium and magnesium (Angelucci et al., 2020). Treatment with deferasirox should be initiated for values higher than 1,000 gL^-1^. The rise in serum ferritin levels is influenced by acute illnesses, chronic inflammation, and fevers, so a transferrin saturation standard of 15–50% may be useful (Eghbali et al., 2019). It is recommended to start with a 20 mgkg^−1^ low dose for infrequent blood exchanges followed by a 30 mgkg^−1^ high dosage for frequent blood exchanges. For the switch from deferoxamine to deferasirox, half of the dose of deferoxamine (DFO) must be administered. If serum ferritin concentrations remain below 500 µgL^-1^, a dose reduction or interruption of treatment should be considered. Generally, medication-associated adverse reactions include elevations in liver enzymes, digestive disorders, and unproductive increases in creatinine (Gottwald et al., 2020).

### 5.4 Deferoxamine (DFO)

A medically acceptable iron-chelating agent called trishydroxamate has proven to be effective in long-term iron chelation therapy, such as in beta-thalassemia (Ansari et al., 2017). The desferrioxamine binding of Fe (III) is highly exclusive, and removes Fe slowly and slowly from hemosiderin and ferritin, except myoglobin, hemoglobin, oxidases, or cytochromes (Yan et al., 2018). DFO is not administered orally due to its poor oral absorption capacity and pharmacokinetics. It is recommended to only administer intramuscularly and with continuous intravenous infusions (Jones et al., 2020). DFO is transported away from the plasma readily with a biological half-life of 5–10 min, which prompted extended subcutaneous infusion (Yu et al., 2020). A nocturnal injection of iron results in the excretion of between 20 and 50 mg of iron daily through the feces and urine. Consequently, it can put a damper on subsequent iron accumulation, thus reducing its storage (Umemura et al., 2017). Even though pediatric patients benefit from these therapies, the infusions are painful, time-consuming, and expensive. Hypotension, abdominal pain, nausea, diarrhea, and vomiting due to chronic treatment are some of the side effects.

### 5.5 Deferiprone

Patients with inadequate response to other iron chelating agents may find Deferiprone a better alternative. In liver tissue culture studies, chelator ratios lower than iron concentrations confirmed an increase in DNA oxidation with the drug. Dosages between 25 mgkg^−1^ and 100 mgkg^−1^ are recommended orally thrice daily (Binding et al., 2019). When deferoxamine is not available, it is considered the second-line treatment in primary beta thalassemia patients. In order to treat severe left ventricular dysfunction successfully, a combination of deferiprone and deferoxamine is administered. Side effects include gastrointestinal agranulocytosis or neutropenia and arthralgia (Olivieri et al., 2019).

There are different ways to compare the costs of deferasirox and deferiprone therapy: the cost of medicine, the cost of adverse effects associated with treatment, and the cost of laboratory tests. So, deferasirox is the top choice followed by both deferiprone and deferoxamine in second and third place, respectively (Wahidiyat et al., 2018). However, deferasirox treatment is favored over traditional deferoxamine therapy according to many recent studies (Eastin and Eastin, 2019).

## 6 Hyperferritinemia as a part of COVID-19

During the early 2020 s, a flare-up of the SARS-CoV-2 virus developed into a pandemic risk. COVID-19 disease is characterized by fatigue, fever, dry cough, lung involvement, and pneumonia (Wang et al., 2020). A proportion of 14% can have serious problems with dyspnea, hypoxemia, and tachypnea, as well as lung parenchyma with 50% pulmonary infiltrates within 24 to 48 h in more serious cases. Septic shock, cardiac arrest, respiratory failure, and organ failure of more than one organ can be life-threatening in less than 5% of cases (Zhou et al., 2020).

Recent research suggests that an excessive immune response, known as cytokine storm, characterized by lymphopenia, chronic high fevers, hyperferritinemia, and elevated interleukin levels, caused death in many COVID-19 patients (Ehrenfeld et al., 2020). An unfortunate side effect of severe infection looks like the cytokine storms demonstrated in other inflammatory conditions, such as MAS, severe viral infections, sepsis, and graft-versus-host disease (Burugu et al., 2020).

As a proinflammatory mediator, ferritin could instantly trigger the cytokine storm as an exogenous mediator (Ruan et al., 2020). Hypoferritinemia, therefore, plays a possible pathogenic role in the cascade of COVID-19 infection. Several studies indicate that COVID-19 mortality is likely caused by virally-driven hyperinflammation, which appears to be a significant indicator of severity and prognosis (Belmont and Kwiatkowski, 2017, Carcillo et al., 2017). Inflammation resulting in hyperferritinemia can result in admission to the intensive care unit and high mortality rates (Du et al., 2020). This indicates the need to identify high-risk patients to guide therapeutic intervention to manage inflammation. A higher ferritin value is more likely to be associated with hemophagocytic lymphohistiocytosis in patients with COVID-19 (Eloseily et al., 2020), and ones with attenuated lung lesions are more likely to have this condition (Eloseily et al., 2020).

Having a ferritin level more than 700 ngmL^−1^ could alert clinicians to more work-up required to diagnose cytokine storm syndrome, allowing them to consider therapeutic approaches without delay (Chen et al., 2020). In a study of 21 patients, Chen *et al.* (2019) reported that serum ferritin levels were greater than 800 gL^-1^ in 11 patients diagnosed with COVID-19 serious infection. In another retrospective study of 191 patients, 96% of non-survivors had serum levels over 300 gL^-1^ (Zhang et al., 2021). Developing cytokine storm syndrome, needing invasive ventilation, and dying in hospital are more likely in patients with low iron and D-dimer values initially, but increasing over time (Metcalf et al., 2020). The need for ventilation and death are low when initial ferritin and D-dimer levels are high and subsequent time-spaced repeated values are stable or decreasing (Qeadan et al., 2021). In certain susceptible patient groups, immunological and physiological feedback mechanisms may be different during SARS-CoV-2 infections, since genetic susceptibility factors may influence cytokine production (Qeadan et al., 2021). As many countries are experiencing a second or possibly a third wave of COVID-19, repeated measurement of serum ferritin and D-dimer will support the identification of cases in which a cytokine storm is likely to develop and which will require intensive care (Times of India, 2021a).

## 7 Second wave of COVID-19 in India

It has been most tragic to witness a more aggressive second COVID-19 wave in India after completing a time course to control the disease in 2020 and launching a vaccination drive in 2021. With 79 cases of COVID-19 resuscitated since February 2021, Delhi, Maharashtra, Uttar Pradesh, Tamil Nadu, Kerala, and Karnataka are the states with the highest number of deaths. In the initial phase of the second wave, the nation recorded 3.9 lakh new cases, or 47% of what was reported by the World Health Organization recently (Cherian et al., 2021). The existence of new mutated variants, including B.1.36 from southern states and B.1.617 from Maharashtra, as well as 3 global variants, has been observed worldwide (Chen et al., 2020).

### 7.1 Mucormycosis

The progression of COVID-19 infection was influenced by many factors, including preexisting comorbidities, diabetes mellitus, use of immunosuppressants, risk of nosocomial infections, and systemic immune modifications. As a result of immune dysregulation, COVID-19 is associated with a significant incidence of bacterial and fungal invasion. Monoclonal antibodies or corticosteroids or wide-spectrum antibiotics are commonly used in the armamentarium against COVID-19 mitigation, and potential side effects may include causing exacerbation of preexisting fungal infections (John et al., 2021).

Worldwide, several cases of COVID-19-associated mucormycosis (CAM) have been reported. We do not yet know the underlying mechanism that links mucormycosis to COVID-19. Other factors, such as diabetic ketoacidosis, glucocorticoids consumption, deteriorating blood glucose control, viral-induced lymphopenia, and hyperferritinemia and acidosis, have been implicated in causating CAM (Times of India, 2021b). In India there were over 5000 cases and 126 casualties, leading to various states declaring an epidemic (Revannavar et al., 2021). Mucormycosis is a fungus-related angioinvasive disease that is caused by Rhizopus; Absidia, and Mucor. In a female patient diagnosed with CAM and without diabetic ketoacidosis, the serum ferritin level was 180.2 mg/dL (reference range for women is 13–150ngmL^−1^; 30–400ngmL^−1^ for men).

In a patient with early-onset diabetes mellitus, COVID-19 was associated with rhinoorbital-cerebral mucormycosis and ketoacidosis (Li et al., 2020). Patients with COVID-19 have been observed to exhibit ketoacidosis and ketonemia, despite the absence of diabetes (Islam et al., 2021). Ketoacidosis disrupts phagocytosis and promotes iron dislocation from transferrin, which leads to the proliferation of this fungal pathogen . In addition, iron and zinc excessively used as immunity boosters may cause free iron overload (Jiang et al., 2021).

## 8 Impact of genomics on therapeutic perspectives

**Fig. 1:**
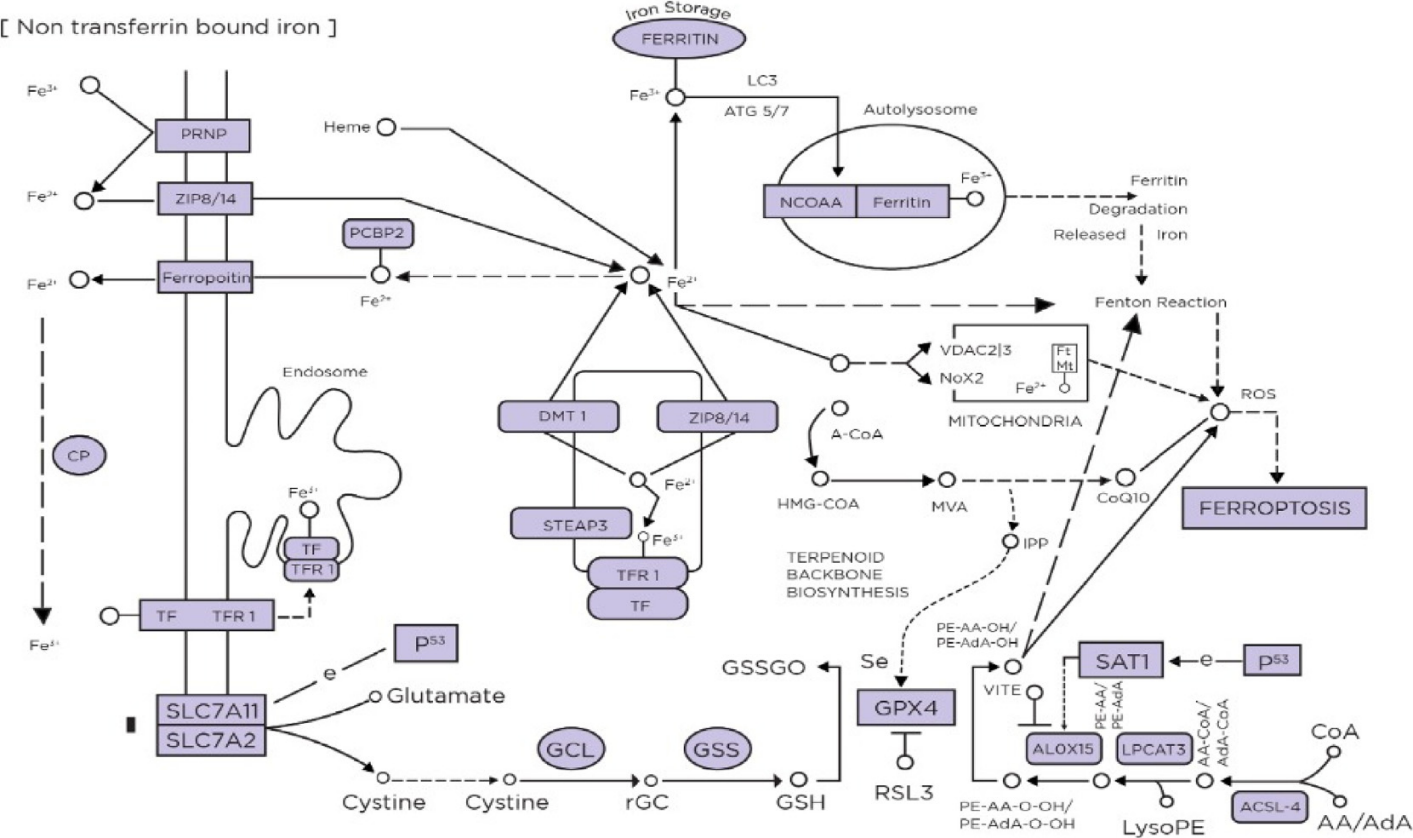
The clinical use of iron chelation therapy for COVID-19 infection. A ferroptosis pathway regulated by catalytic iron generates ROS, lipid peroxides and DNA damage through Fenton reaction or hydroxylation of PUFA. Upon transferrin receptor stimulation, Fe(III) is reduced to Fe(II) and transferred internally via DMT1. Through glutathione cofactor, GPX4 eliminates lipid peroxides. (PRNP- prion protein, CP- Ceruloplasmin, TfR1- Transferrin Receptor 1, GCL-glutamate-cysteine ligase, GSS-glutathione synthetase, GSH-Glutathione, GPX4- glutathione peroxidase, ROS- reactive oxygen species).

Often referred to as controlled cell death, ferroptosis is a novel regulated necrosis characterized by the formation of many reactive oxygen species [ROS] and massive accumulation of lipid peroxides. Genetically and biologically, it differs from other forms of regulated necrosis or cell death. A reduction in cell volume and an increase in mitochondrial membrane concentration are the mechanisms involved (Tang et al., 2021). This occurs when iron homeostasis is disturbed, causing an excessive accumulation of toxic lipid oxidation products and subsequently impairing membrane structure by damaging the cell's antioxidant capacity. A variety of mechanisms regulate ferroptosis including ROS, lipid and iron metabolism pathways (Fig. 1). By producing toxic ROS through the Fenton reaction, boundless iron contributes to ferroptosis. It is triggered by the oxidation of polyunsaturated fatty acids [PUFAs] that leads to lipid peroxidation (Stockwell et al., 2017).

There is evidence that ferroptosis is a pathogenic mechanism in many diseases, such as metabolic diseases, degenerative diseases like Parkinson's disease, ischemic injury, stroke, renal failure, intracerebral hemorrhage and pulmonary diseases (Dar et al., 2019). Infection accompanied by pulmonary injury is a significant cause of the disease. *Pseudomonas aeruginosa* lipoxygenase stimulates PUFA-PE oxidation and bronchial epithelium ferroptosis in host cells (Amaral et al., 2019). According to a study on ferroptosis-mediated tuberculosis, macrophages infected with *Mycobacterium tuberculosis* exhibit ferroptosis features (Yoshida et al., 2019). Smoking induces ferroptosis, which eventually leads to chronic COPD as a consequence of resulting nuclear receptor coactivator 4 (NCOA4)-directed ferritinophagy. Iron chelating agents significantly alleviate the symptoms of all the conditions listed above (Mou et al., 2019). It is possible to use this process to formulate anticancer drugs that promote cell death in cancerous cells (Tang et al., 2018).

Viral replication requires iron as the main raw material. Increased iron can result in the above mentioned catalystic reaction generating lethal reactive species that cannot be expelled by glutathione peroxidase (GPX4) at reduced concentrations (Yang and Lai, 2020). Consequently, iron-dependent cell death may occur in COVID-19 subjects as a result of iron equilibrium destruction. Several patients infected with SARS-CoV-2 develop ferrometabolic dysfunction, which causes multiorgan inclusion, which has rendered ferroptosis a recent therapeutic focus.

It is hypothesized that COVID-19 and ferroptosis are interdependent. Invading SARS-CoV-2 can cause cytotoxicity to multiple organ systems after incubation. Fe [III] ions in the transferrin receptor are transported into the cell by transferrin and transformed into Fe [II] by divalent metal transporter 1 [DMT1], followed by iron accumulation in the cell. The Fenton reaction results in the formation of lipid ROS when lipid, Iron [II] and hydrogen peroxide combine. It can be inhibited by glutathione with the help of GPX4. COVID-19, however, results in extensive cellular damage from extensive ROS production due to its iron burden (Takashi et al., 2020). By destroying iron overload and limiting lipid peroxide formation, iron depletion therapy using iron chelators proves effective at inhibiting ferroptosis. Ciclopirox (CPX), 2,2′-pyridine, deferasirox and deferoxamine (DFO) prevent ferroptosis by reducing iron accumulation (Chaurasia et al., 2019).

### 8.1 Proteomic interactions

The genetic expression of transferrin and iron metabolism are influenced in relation to hypoxic conditions. Regulation of hypoxia-inducible transcription factor (HIF)-1 is tightly associated with their pathways. This protein contains the HIF-1α subunit as an oxygen-responsive regulator and the HIF-1β subunit, also known as aryl hydrocarbon receptor nuclear translocator (ARNT). When oxygen is abundant, the HIF-1α fractional unit undergoes hydroxylation using iron-associated prolyl-hydroxylases (PHDs), which is inhibited when oxygen is scarce. Therefore, it culminates in aggregation of HIF-1α and its displacement to the nucleus, after which it binds to HIF-1β and activates transcription of HIF-responsive genes. By oxidizing iron from PHDs, iron chelating agents, such as quercetin and deferoxamine (DFO), appear to inhibit the HIF-1α hydroxylation, therefore strengthening the protective effects of iron chelation (Shimizu et al., 2020).

## 9 Cinical use of iron chelators in Covid-19 patients

**Fig. 2:**
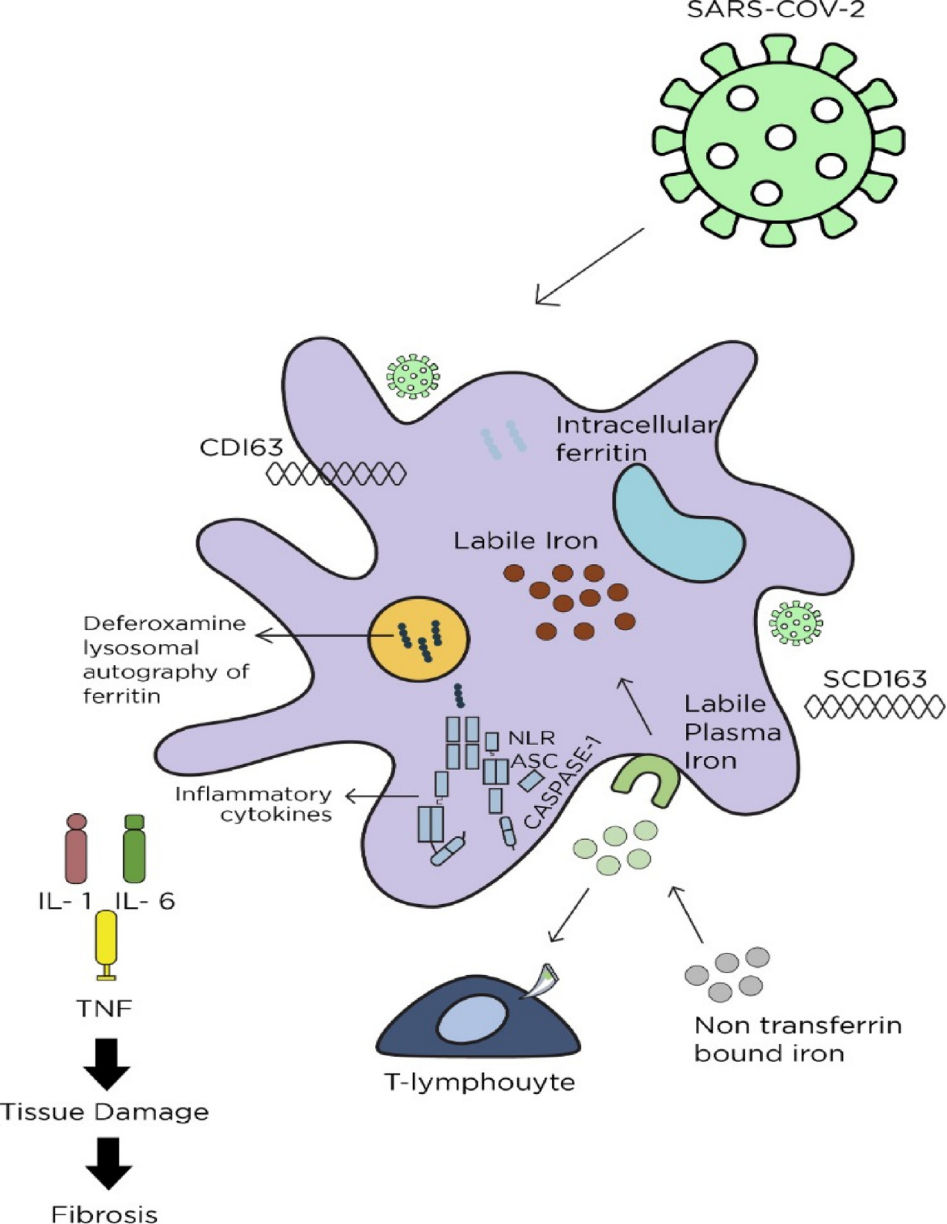
Ferroptosis pathway metabolism: structure and regulation. SARS-CoV-2 activates macrophages, resulting in hyperinflammation and a cytokine storm due to IL-18, IL-6, IL-1, TNF and ferritin levels. Deferoxamine (DFO) induces apoptosis of ferritin in lysosomes and promotes T-cell-mediated activation of IFN- γR2. (SARS-CoV-2- severe acute respiratory syndrome coronavirus 2, IL1- interleukin 1, IL6- interleukin 6, TNF- tissue necrosis factor, CD163-cluster of differentiation 163, SCD163-human soluble cluster of differentiation 163).

As a result of the pathogenic scenario linking inflammation, iron, and infection, it is crucial to seek out a possible therapeutic approach to prevent the onset of fibrosis and cytokine storm that occurs specifically in COVID-19 patients. Thus, iron chelation therapy can be considered as a novel approach to treating hyperferritinemia. In addition to binding with serum-free iron, iron-chelating agents can also explain the potential choice of COVID-19 treatment in other ways. These mechanisms involve iron removal from iron-binding proteins along with downregulation of hepcidin (Liu et al., 2020), and might explain in part why moderate COVID-19 is less likely to cause hyperferritinemia induced by the antiferritin effect (Prakash et al., 2021). The presence of excessive iron in the blood can lead to a response known as cardiac iron toxicity. Oxidative stress from too much free iron can result in a rise in reactive oxygen species [ROS] production. In iron overload management, iron chelation agents such as deferasirox, deferoxamine, and deferiprone have been employed (Fig. 2). These agents have different properties that impact their effectiveness.

The potential for hyperinflammation should be screened initially using laboratory tests such as C-reactive protein (CRP) above the 10-fold reference range, increasing ferritin at 1000 ngmL^−1^, oxygen consumption at 60%, decline in lymphocytes at 1000Χ 10^9^ L^-1^ and declining platelets less than 100 000 × 10^9^ L^-1^. In addition, the progressive deterioration of these parameters indicates an aggressive clinical approach is needed to treat COVID-19. For subjects in the initial stage of COVID-19 infection, oral iron chelators is recommended for 10–14 days (Carota et al., 2021).

Deferasirox, an iron chelator administered orally, has been shown to relieve excessive inflammatory responses and iron overload in both pediatric and adult patients. Among its side effects are skin rashes, gastrointestinal disturbances, and elevated serum creatinine as well as Fanconi syndrome and auditory toxicities (Kandeel and Al-Nazawi, 2020). When deferoxamine is administered intravenously, it is recommended that patients receive 1000 mg initially followed by 500 mg every 12 h until a maximum daily dosage of 6000 mg is tolerated. It is generally recommended that deferoxamine be administered for 24 h (Richard et al., 2016). The dose-related side effects of DFO chronic therapy include renal toxicity, audiology and ophthalmologic complications, as well as certain bacterial infections, particularly *Yersinia enterocolitica.*

The study conducted by Birlutiu *et al*. proposes the use of tocilizumab (TCZ) combined with adjuvant oral deferasirox for cytokine release syndrome in critically ill patients with COVID-19 accompanied pneumonia (Orthomolecular.org). In total, nine (81.81%) of the 11 cases that received deferasirox and TCZ had a favorable outcome, with two unfortunate deaths. Comparing the patient recovery under TCZ with and without an oral iron chelator, deferasirox, showed a slightly unremarkable surge in the adjuvant deferasirox therapy subgroup (80 with 75%) (Orthomolecular.org).

Through the use of antioxidant therapy, COVID-19 treatment can prevent the increase in reactive oxygen free radicals caused by hyperferritinemia. Together with iron chelation therapy, vitamin C can be used as a supportive treatment (Orthomolecular.org). The dose ranged from 10 to 20 g day^_1^ over eight to ten hours. All patients were gradually discharged with quick recovery after the oxygen saturation index was enhanced simultaneously (Orthomolecular.org).

In addition to being a member of the transferrin family, lactoferrin is also a naturally occurring iron chelator since lactoferrin contains more pertinence to binding iron than transferrin. Positively-charged compartments in its structure are ideally suited for Fe [III] binding, which makes it an appropriate treatment option. As well as influencing the immune system, lactoferrin also reduces inflammatory response by regulating ROS and cytokine production. Through its ability to inhibit heparan sulfate proteoglycan binding, LF also shows antiviral qualities (Aboda et al., 2020).

Additionally, Janus kinase [JAK] inhibitors have also been proposed for dealing with severe COVID-19 infections. According to a recent study, baricitinib therapy led to significant reductions in serum cytokines IL-6, TNF, and IL-1b. Baricitinib has been approved for use in combination with antivirals such as remdesivirin in adults and pediatric patients with severe COVID-19 (Bronte et al., 2020). Corticosteroids have been shown to reduce the mortality of severe COVID-19 cases in multiple studies, including the recovery trials. In patients requiring mechanical ventilation, dexamethasone treatment for up to 10 days had a notable effect on 28-day mortality (RECOVERY Collaborative Group, 2021). Treatment with cytokine blockade could lead to a decreased level of IL-6 cytokine, which correlates positively with inflammatory factors including D-dimer, ferritin, and CRP, which can be probable causes of hyperferritinemia (Ibrahim et al., 2006).

COVID-19 patients with elevated serum free iron are at risk of secondary infections like mucormycosis. In a study conducted by Ibrahim et al. (2006), deferiprone was compared to liposomal amphotericin B (LAmB) for controlling mucormycosis in mice with diabetic ketoacidosis (Chauhan et al., 2021). Studies have found that a daily dose of deferiprone or LAmBat of 100 mgkg^−1^ on average improved survival when compared with a placebo. The fungal burden in the brain was reduced by both drugs in comparison to placebo. Deferiprone was found to be as effective as LAmB at restoring survivability of experimental animals (Ibrahim et al., 2006).

In one way or another, every therapeutic option comes with pros and cons. Chauhan et al. (2021) described a male patient aged 18 years who had known beta-thalassemia, presented with gastric perforation due to chelation therapy and coronavirus-2 infections alongside ARDS and ferritin levels of 945 µgL^-1^. As a result of regular blood transfusions and inefficient erythropoiesis, beta-thalassemia can lead to iron overload. A number of randomized control trials have demonstrated the efficacy and safety of iron chelation therapy, but chronic use of deferasirox has been linked to intestinal inflammation and perforation due to mitochondrial swelling in a variety of critical diseases (Henry et al., 2020). SARS-CoV-2 rapid replication in the gastrointestinal epithelium caused the side effect, as evidenced by biopsy and virology analysis of the affected epithelium, which also resulted in bowel perforation and esophageal erosions Henry et al., 2020.

## 10 Conclusion and future perspective

According to our review article, iron and its storage molecule ferritin have a credible pathophysiological role in the human body. Various underlying etiological factors of hyperferritinemia were defined, and chelators were proposed as a way to manage iron overload conditions. Deferoxamine, Deferriprone, and Desferasirox are iron chelating agents that help alleviate hyperferritinemia by scavenging excess iron from the body. With SARS-CoV-2 infection resulting in cytokine release syndrome and hyperferritinemia, iron chelating agents were necessary alone or synergistically with antioxidants, corticosteroids, and monoclonal antibodies. Mucormycosis, one of the secondary complications of COVID-19, is highly dependent on chelating agents such as Deferriprone for survival. The use of chelating agents significantly decreased the need for intermittent mandatory ventilation as well as the mortality rate of critically ill patients, thus serving as an innovative therapeutic strategy for the COVID-19 outbreak.

In the context of the current pandemic scenario, iron homeostasis has to be further explored. It is important to emphasize the necessity of iron chelation therapy in preventing the COVID-19 cytokine storm. Furthermore, we recommend that further experimental studies demonstrate the effect of hyperferritinemia on the cardio-respiratory health and fatality of COVID-19 victims.

Our review article indicates the credible pathophysiological role of iron and its storage molecule ferritin in the physiological process of the human body. Underlying etiological factors for hyperferritinemia were defined in various disease conditions and proposed the use of chelators to manage iron overload conditions. Iron chelating agents like deferoxamine, deferriprone, and desferasirox supports alleviating hyperferritinemia by scavenging excess of iron from the body. The impact of cytokine release syndrome and hyperferritinemia accompanied by the severity of SARS-CoV-2 infection necessitated iron chelating agents alone or synergistically with antioxidants, corticosteroids and monoclonal antibodies. The secondary complications arising from COVID-19 such as mucormycosis, greatly depend on chelating agents such as deferriprone for survival. The utilization of chelating agents progressed to a significant diminishment in demand for intermittent mandatory ventilation likewise the mortality fraction of critically ill patients, thus functioning as an innovative therapeutic strategy in the COVID-19 outbreak.

The relationship between iron homeostasis and COVID-19 must be further elucidated in the current pandemic scenario. And, the necessity of iron chelation therapy should be emphasized for better mitigating cytokine storm associated with COVID-19. We also recommend future experimental studies that demonstrate the consequence of hyperferritinemia on the cardio-respiratory well-being and fatality of COVID-19 victims.

## Declaration of Competing Interest

The authors declare that they have no known competing financial interests or personal relationships that could have appeared to influence the work reported in this paper.
